# Circulating linoleic acid at the time of myocardial infarction and risk of primary ventricular fibrillation

**DOI:** 10.1038/s41598-022-08453-0

**Published:** 2022-03-14

**Authors:** Teresa Oliveras, Iolanda Lázaro, Ferran Rueda, Germán Cediel, Deepak L. Bhatt, Montserrat Fitó, Francisco Madrid-Gambin, Oscar J. Pozo, William S. Harris, Cosme García-García, Aleix Sala-Vila, Antoni Bayés-Genís

**Affiliations:** 1 Department of Cardiology, Heart Institute, Hospital Universitari Germans Trias i Pujol, c/Canyet s/n, 08916 Badalona, Barcelona Spain; 2 Department of Medicine, Universitat Autònoma de Barcelona, Barcelona, Spain; 3 Centro de Investigación Biomédica en Red Enfermedades Cardiovasculares (CIBERCV), Instituto de Salud Carlos III, Madrid, Spain; 4 Cardiovascular Risk and Nutrition–IMIM (Hospital del Mar Medical Research Institute), Dr. Aiguader 88, 08003 Barcelona, Spain; 5 Brigham and Women’s Hospital Heart & Vascular Center, Harvard Medical School, Boston, MA USA; 6 Centro de Investigación Biomédica en Red de la Fisiopatología de la Obesidad y Nutrición (CIBEROBN), Instituto de Salud Carlos III, Madrid, Spain; 7 Signal and Information Processing for Sensing Systems, Institute for Bioengineering of Catalonia (IBEC), Barcelona Institute of Science and Technology, Barcelona, Spain; 8 Fatty Acid Research Institute, Sioux Falls, SD USA; 9 Sanford School of Medicine, University of South Dakota, Sioux Falls, SD USA

**Keywords:** Lipidomics, Cardiology

## Abstract

Primary ventricular fibrillation (PVF) is a major driver of cardiac arrest in the acute phase of ST-segment elevation myocardial infarction (STEMI). Enrichment of cardiomyocyte plasma membranes with dietary polyunsaturated fatty acids (PUFA) reduces vulnerability to PVF experimentally, but clinical data are scarce. PUFA status in serum phospholipids is a valid surrogate biomarker of PUFA status in cardiomyocytes within a wide range of dietary PUFA. In this nested case–control study (n = 58 cases of STEMI-driven PVF, n = 116 control non-PVF STEMI patients matched for age, sex, smoking status, dyslipidemia, diabetes mellitus and hypertension) we determined fatty acids in serum phospholipids by gas-chromatography, and assessed differences between cases and controls, applying the Benjamini–Hochberg procedure on nominal P-values to control the false discovery rate (FDR). Significant differences between cases and controls were restricted to linoleic acid (LA), with PVF patients showing a lower level (nominal P = 0.002; FDR-corrected P = 0.027). In a conditional logistic regression model, each one standard deviation increase in the proportion of LA was related to a 42% lower prevalence of PVF (odds ratio = 0.58; 95% confidence interval, 0.37, 0.90; P = 0.02). The association lasted after the inclusion of confounders. Thus, regular consumption of LA-rich foods (nuts, oils from seeds) may protect against ischemia-driven malignant arrhythmias.

## Introduction

Coronary artery disease (CAD) remains a major health challenge and a top cause of global mortality^1^. Though advances in drugs and device-based therapies have largely reduced complications and improved outcomes for those who experience a myocardial infarction (MI), the rate of primary ventricular fibrillation (PVF) has remained stable over time^2,3^. PVF is a major trigger of out-of-hospital cardiac arrest leading to sudden cardiac death^4^, and patients developing ventricular fibrillation during acute MI are at higher risk of in-hospital mortality^5^. Therefore, novel strategies are needed to prevent and manage acute ischemic PVF.

Experimental research has been instrumental in understanding the arrhythmogenic mechanisms leading to PVF. In this regard, many metabolic and electrophysiological cardiac changes observed in the acute ischemic phase underlie disturbances in voltage-gated channels^6^. A large body of evidence indicates that polyunsaturated fatty acids (PUFA) acylated in the phospholipids constituting the lipid bilayers of cell membranes modulate the activity of voltage-gated channels by either altering the biophysical membrane properties (indirect effect) or binding to the protein upon cleavage from cell membrane phospholipids (direct effect)^7,8^. The finding that dietary fats are readily incorporated into the cell membranes of cardiomyocytes put for the hypothesis that sustained dietary PUFA intake and the ensuing enrichment in cardiac phospholipids might reduce myocardial vulnerability to early PVF in MI^9^.

PUFA include mainly omega-3 (n-3) and omega-6 (n-6) fatty acids. n-3 PUFA, particularly those of marine origin (eicosapentaenoic acid [C20:5n-3, EPA] and docosahexaenoic acid [C22:6n-3, DHA]), have been shown to possess an array of cardioprotective effects^10^. Regarding n-6 PUFA, in particular linoleic acid (C18:2n-6, LA), although sustained intake of LA has been mechanistically linked to an increased low-density lipoprotein oxidation and to the transformation to arachidonic acid (C20:4n-6, AA—a precursor of proinflammatory lipid mediators), there is increasing evidence of the cardioprotective benefits of LA intake within the range advocated by the American Heart Association^11^. Experimental research uncovered a long time ago that replacement of saturated animal fat in the diet with either LA-rich or n-3-rich oils reduced the incidence and severity of arrhythmias occurring in ischemia^9^. However, this notion barely translated into clinical research. We hypothesized that in patients with ST-elevation myocardial infarction (STEMI), cardiac enrichment in specific fatty acids resulting from the consumption of fat-rich foods during the weeks prior to the event would influence the vulnerability of the myocardium to develop PVF. To address this issue, at hospital admission for STEMI, we performed lipidomics in 58 patients who developed PVF and in 116 non-PVF controls matched for cardiovascular risk factors, searching for associations between fatty acid species and incident PVF. Given that routine myocardial biopsy is not safe in the acute phase of STEMI, we determined fatty acidsin serum phospholipids, the status of which changes in parallel with heart phospholipids within a wide range of dietary fats^12^.

## Results

**Figure 1:**
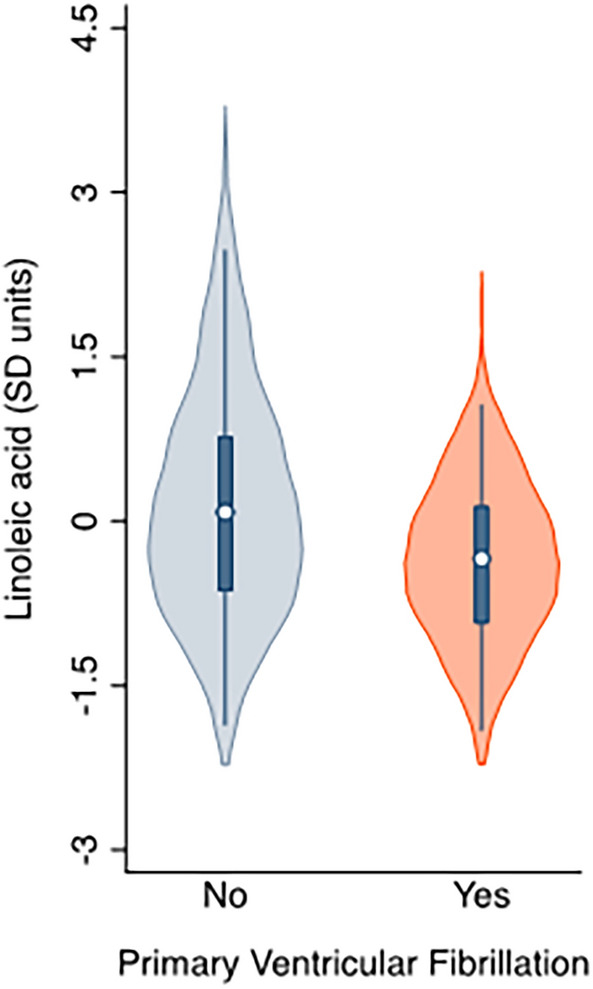
Serum phosphatidylcholine linoleic acid proportion by diagnosis of ventricular fibrillation.

**Figure 2:**
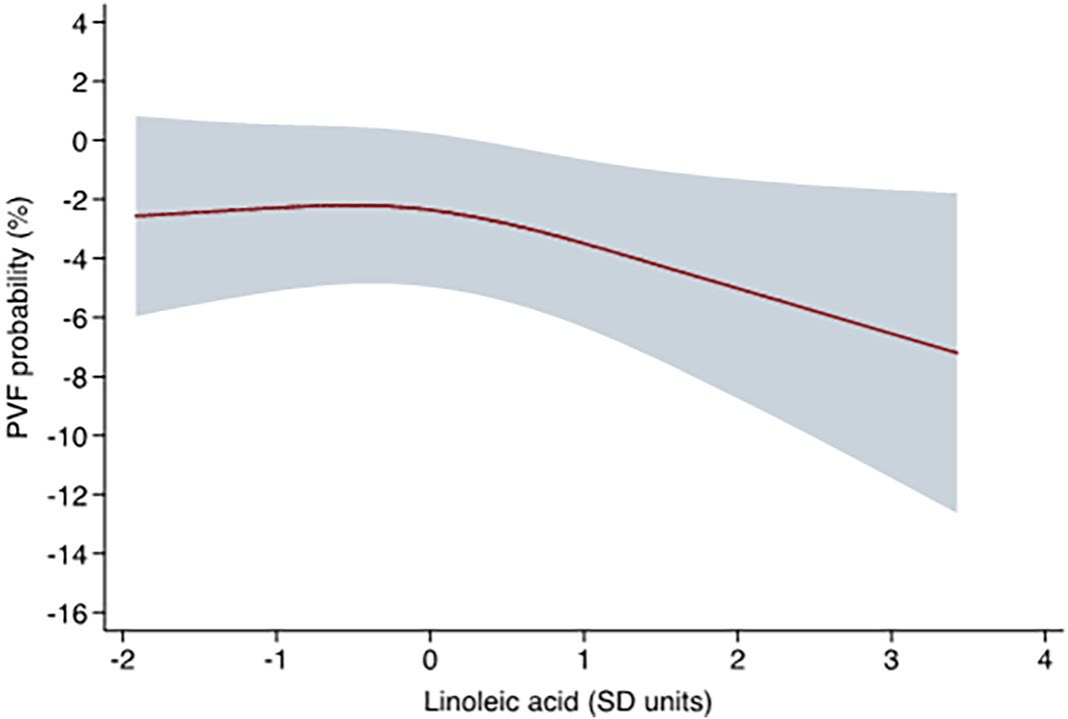
Adjusted predictions with the 95% confidence interval for the relationship between standardized linoleic acid (C18:2n-6) values and the occurrence of primary ventricular fibrillation (PVF). Standardized C18:2n-6 values were modeled by restricted cubic splines. All predictors were set to their mean values.

**Figure 3:**
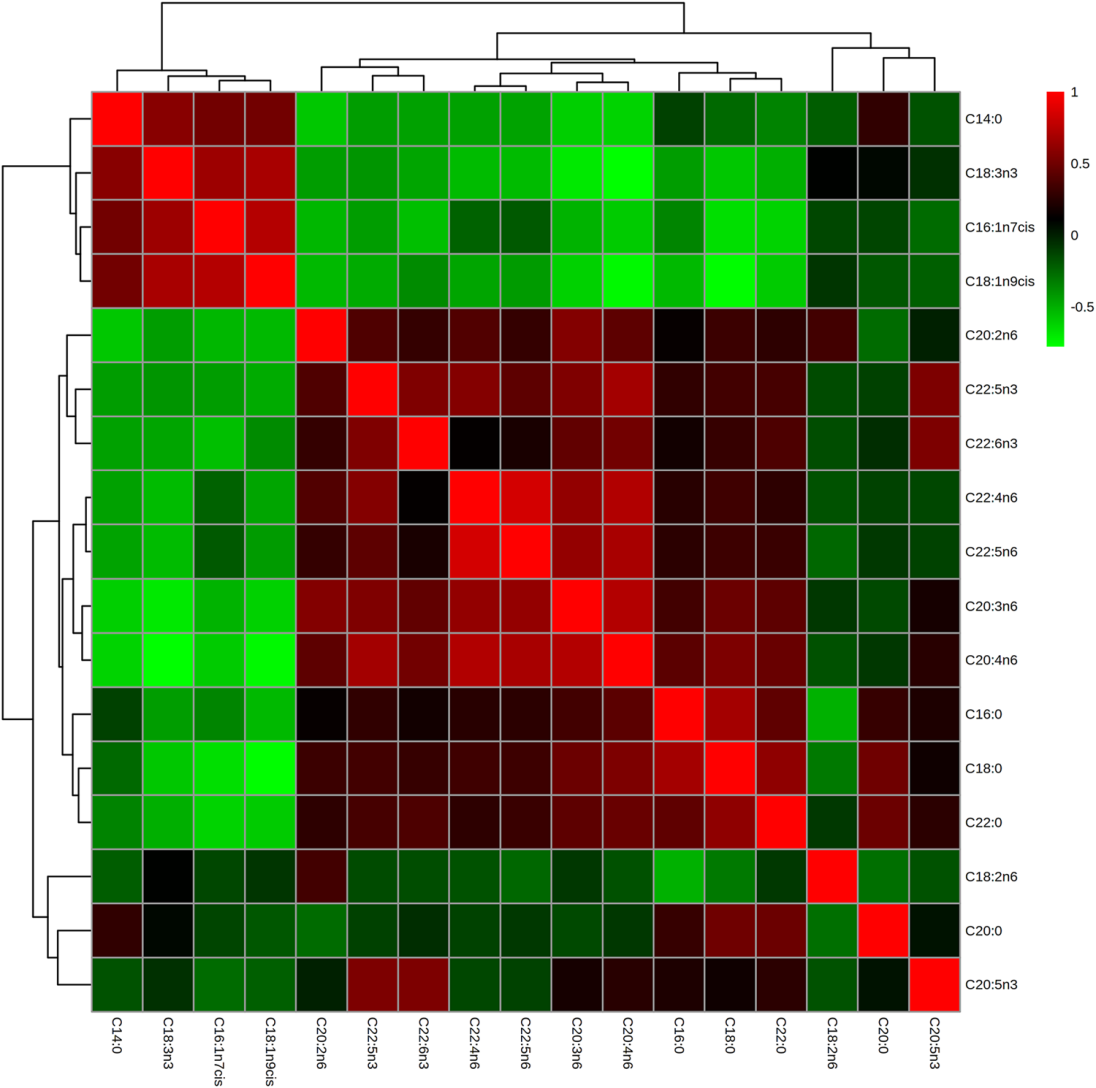
Spearman correlation coefficients in all available fatty acid species in serum phospholipids.

Table 1 provides the participants' characteristics by study group. Per the study design, we found no differences in age, sex, smoking status, or prevalence of treated dyslipidemia, diabetes mellitus or hypertension. We neither observed differences in plasma concentrations of total cholesterol, triglycerides or other known risk factors for PVF as advanced Killip-Kimball class or anterior infarct location. When comparing serum phospholipids status of main fatty acids at hospital admission by study group, significant differences were restricted to LA, with PVF patients showing a lower compared with matched controls (nominal P value = 0.002; false discovery rate [FDR]-corrected P value = 0.027; Table 2 and Fig. 1). When assessing the odds ratios (OR) for the prevalence of PVF associated with this PUFA, the unadjusted model (model 1) showed that each 1-standard deviation (SD) increase in the proportion of LA was related to a significant reduction (42%) in the prevalence of PVF (OR, 0.58; 95% confidence intervals [CI], 0.38 to 0.88; P = 0.01). The observed association remained essentially unchanged after the inclusion of confounders (Model 2, OR, 0.58; 95% CI, 0.37 to 0.90; P = 0.02). A non-linear pattern between LA and the odds of PVF was confirmed in an analysis using restricted cubic spline models that indicated a nadir of risk at 0 SD and decreasing odds of PVF at higher levels of LA (Fig. 2). Finally, the Spearman correlation coefficients for fatty acid species are shown in Fig. 3. We observed that n-6 PUFA were segregated in two groups. While ≥ 20-carbon species showed strong direct interrelationships (Spearman correlation coefficients ranging from 0.29 to 0.84), LA showed marginal and inverse associations with their longer-chain n-6 counterparts (Spearman correlation coefficient between LA and AA = − 0.17).

**Table 1:** Baseline characteristics of primary ventricular fibrillation (PVF) cases and matched controls.

Variable	PVF cases (n = 58)	Non-PVF controls (n = 116)	P-value
**Demographics**			
Age, years	59.9 ± 13.1	59.9 ± 12.6	0.99
Female	7 (12.1)	14 (12.1)	1.00
**History**			
Smoking	33 (56.9)	65 (56.0)	0.91
Hypertension	29 (50.0)	58 (50.0)	1.00
Diabetes mellitus	12 (20.7)	25 (21.6)	0.89
Dyslipidemia	35 (60.3)	64 (55.2)	0.52
Cerebrovascular disease	4 (6.9)	9 (7.8)	0.84
Myocardial infarction	8 (13.8)	8 (6.9)	0.14
PCI	8 (13.8)	10 (8.6)	0.29
CABG	0 (0)	0 (0)	–
**Physical examination**			
Killip-Kimball class III–IV	6 (10.3)	5 (4.3)	0.12
BMI, kg/m^2^	27.7 ± 5.2	27.3 ± 4.1	0.53
Anterior infarct location	29 (48.3)	46 (38.7)	0.22
**Angiography, ≥ 70% stenosis**	58 (100)	114 (98.3)	0.48
0	2 (3.4)	0 (0)	0.04
1	35 (60.3)	60 (51.7)	0.28
2	9 (15.5)	28 (24.1)	0.19
3	12 (20.7)	26 (22.4)	0.79
Successful primary PCI	55 (94.8)	106 (91.4)	0.42
LVEF, %	48.4 ± 10.6	51.8 ± 9.5	0.03
**Laboratory results**			
Hemoglobin, g/dL	13.4 ± 1.6	13.0 ± 1.6	0.14
eGFR, mL/min/1.73 m^2^	76.7 ± 30.6	86.4 ± 31.0	0.05
Total cholesterol, mg/dL	164.8 ± 36.0	175.9 ± 37.3	0.07
Triglycerides, mg/dL	114 (91–158)	112 (57–144)	0.49

Data are presented as n (%), mean ± standard deviation, or median (interquartile range: Q1–Q3). BMI, body mass index; CABG, coronary artery bypass graft; eGFR, estimated glomerular filtration rate; LVEF, left ventricular ejection fraction; PCI, percutaneous coronary intervention.

P value obtained by χ^2^ test, Student’s t test or Wilcoxon rank sum test, as appropriate.

**Table 2:** Proportion of fatty acids in serum phospholipids in primary ventricular fibrillation (PVF) cases and matched controls at hospital admission for STEMI.

Fatty acid	PVF cases (n = 58)	Non-PVF controls (n = 116)	Nominal P-value	FDR-corrected P-value	Cohen’s d
C14:0	3.26 ± 2.09	2.90 ± 2.20	0.151	0.802	− 0.17
C16:0	29.06 ± 3.62	28.55 ± 4.00	0.379	0.802	− 0.13
C16:1n-7*cis*	1.48 ± 1.21	1.33 ± 0.92	0.273	0.802	− 0.15
C18:0	13.44 ± 3.62	12.96 ± 3.06	0.487	0.802	− 0.14
C18:1n-9*cis*	18.87 ± 6.65	18.16 ± 7.07	0.418	0.802	− 0.10
C18:2n-6	17.08 ± 3.27	19.20 ± 4.53	0.002	0.027	0.51
C18:3n-3	0.28 ± 0.18	0.31 ± 0.42	0.513	0.802	0.06
C20:0	0.14 ± 0.08	0.13 ± 0.07	0.605	0.802	− 0.11
C20:2n-6	0.29 ± 0.08	0.31 ± 0.09	0.250	0.802	0.19
C20:3n-6	2.56 ± 0.99	2.67 ± 1.11	0.752	0.802	0.10
C20:4n-6	8.62 ± 3.00	8.61 ± 3.35	0.668	0.802	− 0.01
C20:5n-3	0.60 ± 0.37	0.56 ± 0.37	0.366	0.802	− 0.09
C22:0	0.10 ± 0.05	0.12 ± 0.13	0.618	0.802	0.14
C22:4n-6	0.31 ± 0.13	0.31 ± 0.12	0.804	0.802	− 0.06
C22:5n-3	0.22 ± 0.09	0.22 ± 0.10	0.610	0.802	− 0.06
C22:5n-6	0.55 ± 0.16	0.54 ± 0.17	0.587	0.802	− 0.02
C22:6n-3	3.14 ± 1.10	3.14 ± 1.23	0.755	0.802	0.00

Data are presented as mean ± standard deviation. Nominal P-values were assessed by Student’s t test, and the Benjamini–Hochberg procedure was applied on nominal P-values to control the false discovery rate (FDR).

## Discussion

In this case–control, prospective study enrolling a large cohort of STEMI-driven PVF patients and matched non-PVF STEMI controls, we determined the fatty acid status of serum phospholipids at hospital admission for STEMI. These objective lipidomic biomarkers mirror not only dietary intake during the previous weeks, but also the fatty acid status in inner membranes, including cardiomyocytes. We found that a lower prevalence of PVF was associated with increasing levels of LA, an essential n-6 fatty acid naturally found in nuts (such as walnuts, pine nuts, pistachios and almonds) and seed oils (such as canola, corn, safflower, soybean, and sunflower oils).

Our findings are clinically relevant in two ways. First, we provide novel clinical evidence of a modifiable lifestyle (dietary) factor that is related to a lower risk of PVF, an endpoint that is a major contributor to short-term mortality in STEMI survivors. PVF is difficult to predict, and occurs at persistently high rates despite advances in the era of PPCI. Therefore, identifying any easily accessible and safe strategy to reduce the risk for PVF in STEMI patients is needed. Second, because LA in serum phospholipids reflects dietary levels of this essential fatty acid^12,13^, we suggest that intake of this n-6 PUFA may protect against PVF. This observation was repeatedly reported in animal models^11^ and may be mechanistically explained by changes in membrane cells^14^ upon incorporation of dietary LA into the phospholipids, as occurs with other dietary PUFA^12^. Notably, because LA can be transformed into AA, a substrate of many proinflammatory lipid mediators, dietary LA is widely perceived to promote inflammation, contributing to cardiovascular disease. Although LA can be converted to a few inflammatory and vasoconstrictory lipid mediators^15^, there is a growing body of evidence for the potential cardio-metabolic benefits of LA^16^, including a recent landmark study pooling data from 30 prospective cohorts, which reported that higher in vivo circulating and tissue levels of LA were associated with lower risk of major cardiovascular events^17^. Our data contribute to countering the demand to remove LA from the diet based on its proposed harmful effects. Indeed, seed oils that used to be rich sources of LA, such as safflower and sunflower oils, have now been hybridized to substantially remove LA. According to a recent modeling experiment, this shift may be placing the population (children in particular) at increased risk of a deficiency in essential fatty acids, including LA^18^. Studies such as ours may help build a firmer evidence base for the benefits of LA, which will hopefully slow (or even reverse) these well-intentioned but, in our view, misguided efforts to remove LA from the diet.

Interestingly, we failed to find significant associations for marine-derived n-3 fatty acids. Though a large body of observational evidence exists on the benefits of dietary EPA and DHA against sudden cardiac death, the issue of whether dietary EPA and DHA may protect against PVF in the setting of STEMI remains unsettled^19^, with randomized controlled trials conducted in patients with chronic myocardial scars wearing implantable cardioverter defibrillators who already have a history of ventricular arrhythmia^20–22^. Further research is warranted to clarify effects of dietary n-3 PUFA on PVF.

This study had several limitations. First, its observational nature precludes establishing causality between circulating LA (or dietary LA intake) and prevention of PVF in the setting of STEMI. Such a link could only be confirmed by a randomized controlled trial of dietary supplementation with LA before the occurrence of STEMI—which practically speaking would be challenging to design. Second, we used the fatty acid profile of serum phospholipids. Although the use of this lipidomic-based objective biomarker of long-term dietary fatty acid intake circumvents the disadvantages of self-reported dietary data (i.e., food diaries and food frequency questionnaires), the fatty acid profile of serum phospholipids does not reflect long-term intake as accurately as the fatty acids in adipose tissue or red blood cells. Third, dietary LA in Mediterranean populations is largely supplied by nuts and seeds^23^, which contain many cardioprotective bioactive agents in addition to PUFA^24^. Therefore, we cannot rule out the parent foods themselves being the actual protective agent and LA just a marker of their consumption. Finally, because some potential health-related confounding variables (i.e., socioeconomic status, education, adherence to Mediterranean diet) were not available, we could not exclude the possibility that uncaptured environmental factors may have influenced or caused the observed association.

In conclusion, we identified an association of elevated LA in serum phospholipids at the time of STEMI with a lower risk of PVF. Thus, sustained consumption of sources rich in LA may reduce the risk of ischemia-driven cardiac arrest. Our results, which concur with experimental data and suggested membrane-based benefits ascribed to dietary LA, contribute to dispel the notion that the entire n-6 family of fatty acids promote cardiovascular disease.

## Methods

### Study design and population

The study was a post-hoc, nested case–control study of participants included in the Ruti-STEMI biomarkers study between February 23, 2011, and January 30, 2016. The study included prospectively consecutive patients with STEMI in a stable and well-defined geographic area of approximately 850,000 inhabitants in the northern metro area of Barcelona in Catalonia, Spain, within the Codi IAM primary percutaneous coronary intervention (PPCI) network. For the current analysis, only patients having a blood sample available for fatty acid measurement^25^ were considered (n = 944).

Cases (n = 58) consisted of all individuals diagnosed with PVF during the acute phase of STEMI. Controls were STEMI patients who did not develop PVF during the first 48 h after symptom onset. They were selected by matching two controls with each case using the following criteria: age (± 3 years), sex, smoking status, dyslipidemia, diabetes mellitus, and hypertension. A total of 116 controls were matched to cases (Supplementary Fig. S1).

The STEMI diagnosis was established according to the current universal definition of MI at the time of the study^3^, which included chest pain and electrocardiogram showing ST-segment elevation in two or more contiguous leads (minimum 0.1 mV in the frontal leads and 0.2 mV in the precordial leads) or with new-onset left bundle branch block^3^. Baseline demographics and clinical data were recorded during hospital admission.

In our present analysis, patients were classified according to whether they developed PVF in the first 48 h and included in-hospital (if occurring within hospital facilities before, during, or after PPCI up to 48 h after MI diagnosis) and out-of-hospital PVF (within the EMS system) when arriving alive to the hospital. Events beyond 48 h likely include secondary ventricular fibrillation in a dysfunctional myocardium and were not considered here.

The study was conducted in accordance with the guidelines of the Declaration of Helsinki and was approved by the local Ethics Committee (The Ethics Committe of the Clinical Investigation of Germans Trias i Pujol Hospital, reference EO-11-061). Patients or their representatives provided written informed consent.

### Laboratory measurements

Blood samples were obtained from veno-puncture soon after admission and within 12 h after symptom onset. Samples were processed in a central laboratory to measure biomarkers. Serum was stored at − 80 °C until fatty acid analysis. Total serum lipids were extracted with 2 mL of chloroform/methanol (2:1 v/v). After shaking, the mixture was centrifuged (5 min at 3500 rpm at room temperature), and the organic phase was transferred to a borosilicate glass tube and evaporated to dryness under N_2_ at 30ºC. Phosphatidylcholine was isolated by solid-phase extraction as described in Burdge et al.^26^. Briefly, dried serum lipid extract was redissolved in dry chloroform and applied to an aminopropyl silica column (Sep-Pak Vac NH_2_ cartridges, 100 mg packed silica per 1 mL, Waters) under gravity. After eluting neutral lipid fractions (namely cholesteryl esters and triglycerides), we collected phosphatidylcholine upon elution with a mixture of dry chloroform and methanol. The fraction was evaporated to dryness under N_2_ at 30 °C, and fatty acids were then hydrolyzed and methylated by acidified methanol^26^. Briefly, 1 mL of dry methanol containing H_2_SO_4_ (2%) was added to the tube, which was capped and placed into a block heather (50 °C) for 2 h. After cooling, 80 µL of a solution containing K_2_CO_3_ and 500 µL of n-hexane were added. The tubes were shaken for 1 min, and then centrifuged for 5 min at 3500 rpm at room temperature to separate the layers. The hexane layer was transferred into an automatic injector vial equipped with a glass insert of 300 µL. Fatty acid methyl esters were injected to an Agilent HP 7890 Gas Chromatograph equipped with a 30 m × 0.25 µm × 0.25 mm SupraWAX-280 capillary column (Teknokroma, Barcelona, Spain), an autosampler, and flame ionization detection. Gas-chromatography was run using an optimized temperature program, as follows: the program started at 50 °C, held for 2 min and increased to 220 °C at a rate of 4 °C/min. Helium was used as a carrier gas (30 psi, constant pressure mode). Temperature of injector and detector was 260 °C. The amount of each fatty acid is expressed as a percentage of the total amount of fatty acids.

### Statistical analysis

Summary statistics including counts and percentages are provided for categorical variables and both means with standard deviations and medians with interquartile ranges are provided for continuous variables. Categorical variables were compared using the χ^2^ test. Continuous variables were compared using the Student’s t test or Wilcoxon rank sum test. Differences between PVF cases and matched controls for log-transformed fatty acids of serum phospholipids (C14:0, C16:0, C16:1n-7*cis*, C18:0, C18:1n-9*cis*, C18:2n-6, C18:3n-3, C20:0, C20:2n-6, C20:3n-6, C20:4n-6, C20:5n-3, C22:0, C22:4n-6, C22:5n-3, C22:5n-6; C22:6n-3) were assessed by Student’s t test with effect sizes calculated by Cohen’s d-test, and the Benjamini–Hochberg procedure was applied on nominal P-values to control the FDR^27^. An FDR-corrected P value of < 0.05 was considered statistically significant. The ORs with confidence intervals CIs for PVF associated to PUFA surviving the adjustment by the FDR procedure were assessed by conditional logistic regression model. In addition to an unadjusted model (model 1), a model adjusted for left ventricular ejection fraction, Killip-Kimball class and anterior infarct location (y/n) was also constructed (Model 2). ORs reflecting a change of one SD in a given fatty acid and the CIs are reported. Finally, the interrelationship between selected serum phospholipids PUFA was assessed using the Spearman rank correlation and plotted in heatmap format.

Differences were considered significant if P < 0.05. All analyses were performed in STATA V.13.0 (StataCorp, College Station, TX) or R Software version 4.0.3^28^.

## Supplementary Information

Supplementary Figure S1.

## Data Availability

Data described in the article will be made available upon request pending application and approval.
