# Supplementary figures and images for: Circulating linoleic acid at the time of myocardial infarction and risk of primary ventricular fibrillation
Source: Sci Rep. 2022 Mar 14;12:4377. doi: 10.1038/s41598-022-08453-0 (PMC8921268; doi:10.1038/s41598-022-08453-0)

**Supplementary Figure (S1).** Flow-chart of the study.


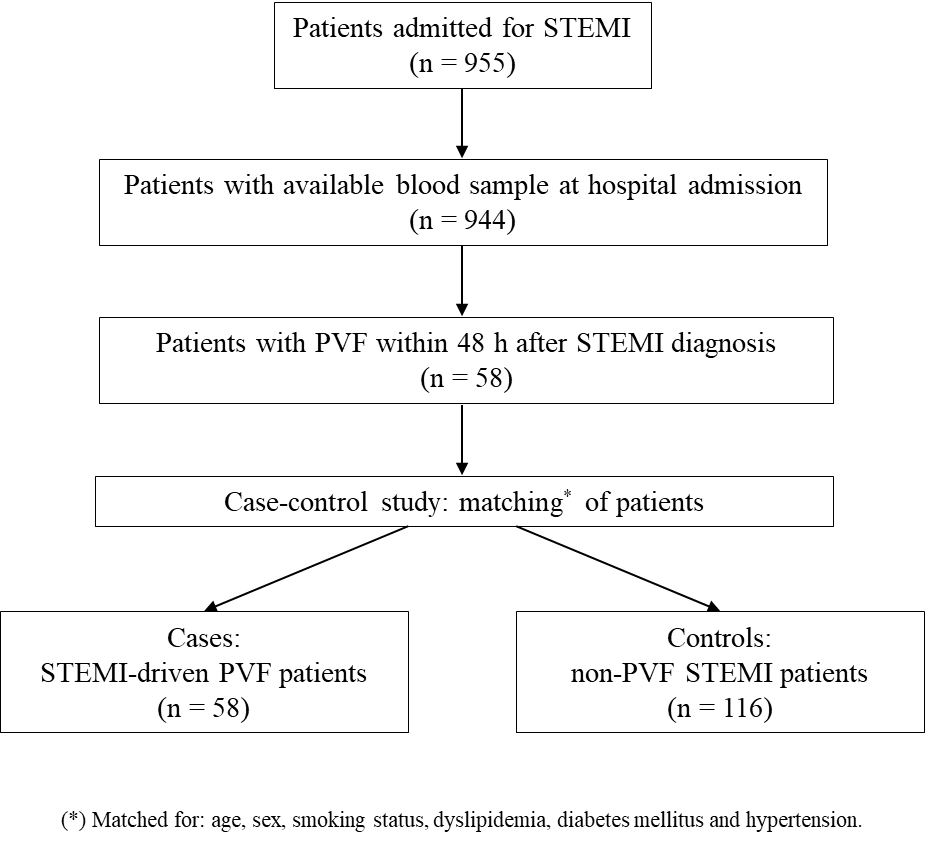

Supplement: Supplementary file 1 — Supplementary Figure S1. [file 41598_2022_8453_MOESM1_ESM.docx]
